# Termite Species Distribution and Flight Periods on Oahu, Hawaii

**DOI:** 10.3390/insects8020058

**Published:** 2017-06-05

**Authors:** Reina L. Tong, J. Kenneth Grace, Makena Mason, Paul D. Krushelnycky, Helen Spafford, Maria Aihara-Sasaki

**Affiliations:** College of Tropical Agriculture and Human Resources, University of Hawaii at Mānoa, 3050 Maile Way, Gilmore Hall 310, Honolulu, HI 96822, USA; masonmak@hawaii.edu (M.M.); pauldk@hawaii.edu (P.D.K.); hspaffor@hawaii.edu (H.S.); aiharasa@hawaii.edu (M.A.-S.)

**Keywords:** *Coptotermes formosanus*, *Incisitermes immigrans*, swarming, citizen science

## Abstract

Termites are economically-important structural pests, costing residents of Hawaii over $100 million annually. On Oahu, the last published termite swarming survey occurred from 1969 to 1971, and the last termite hand-collection survey occurred from 1998 to 2000. To contribute data on termite occurrences on Oahu, a light-trap survey took place from February 2011 to September 2012, and a hand-collection survey occurred from September to November 2012. Formosan subterranean termite, *Coptotermes formosanus* Shiraki, swarming was compared over the duration of the study, finding peak swarming in May 2011. *C. formosanus* alate activity density was regressed with environmental factors, finding a negative correlation with average wind speed and a positive correlation with average rainfall. *Coptotermes gestroi* (Wasmann) alates were observed in April, June, and July 2011 and in June 2012. Four species of termites were found in the hand-collection survey of 44 sites: *Incisitermes immigrans* (Snyder) (*n* = 8/44), *C. formosanus* (*n* = 2/44), *Cryptotermes cynocephalus* Light (*n* = 1/44), and *Neotermes* sp. (*n* = 1/44). This study contributes to distribution data for termite species on Oahu and records alate activity for two important termite pests.

## 1. Introduction

Termites (Blattodea; formerly Isoptera) are social consumers of cellulose and lignocellulose found in dead wood, grass, microepiphytes, leaf litter, and sometimes cultivated fungi [1]. About 3000 species of termites have been described, most having a tropical and temperate distribution [1]. They are estimated to process between 50% and 100% of dead plant biomass in the tropics [1]. In tropical and subtropical areas, termites account for 10% of animal biomass, and for 95% of soil insect biomass [2]. Termite presence and activities create favorable conditions for primary producers, including maintaining soil pH, increasing water retention, mediating decomposition and nutrient cycling, and creating surface areas suitable for microbial colonization [2,3]. They are also an important food source for a variety of animals, such as birds, amphibians, reptiles, and mammals, including humans [4].

However, some species of termites also feed on plant material and lumber used by humans, necessitating expensive repairs, prevention, and control efforts [5]. Termites may also damage non-cellulosic materials, including electrical and telephone wiring, cables, dams, and farming equipment [2,6]. Worldwide termite control and repair costs are estimated at $40 billion, with 80% of those costs attributed to termites from the family Rhinotermitidae [5].

### 1.1. Termites in Hawaii

Oahu is the third-largest and most populous island of Hawaii, with about 80% of the state’s population [7]. Two major mountain chains span the island: the Koolau Range on the eastern coast (~500 to 950 m high by ~72 km long) and the Waianae Range on the western coast (~470 to 1200 m by ~56 km long) [7]. Oahu has two primary physiographic zones, windward (W) and leeward (L), with higher rainfall on the windward side [8]. Due to the small variation in solar radiation, buffering of the ocean, and the effect of trade winds, Oahu has mild temperatures [8]. Oahu has a colder, wetter season from October to April and a warmer season from May to September [8].

Ripperton and Hosaka [9] characterized five major vegetation zones primarily to describe areas with similar climate, soils and vegetation types for use in agriculture in Hawaii (Table 1). Vegetation zone is highly influenced by rainfall. However, zones sometimes have lower or higher average annual rainfalls than listed because of wind and altitude, which affect evaporation and water retention [9].

Eight species of termites from three families are found in Hawaii (Table 2). Of the 183 termite pest species in the world, Hawaii is home to two of the most economically important termite pests, the Formosan subterranean termite, *Coptotermes formosanus* Shiraki, and the Asian subterranean termite, *Coptotermes gestroi* (Wasmann), as well as the most damaging drywood termite pest, the West-Indian drywood termite, *Cryptotermes brevis* (Walker) [5]. All of these species are introduced to Hawaii. Of the eight species found in Hawaii, only one, *Neotermes connexus* Snyder, the forest tree termite, is not found on the current list of twenty-eight invasive termites worldwide [10]. Termite control and repair costs residents of Hawaii over $100 million annually [11], which has likely increased since 1990 [12].

Originating in China, the Formosan subterranean termite, *C. formosanus*, was officially recorded on Oahu in 1913, but was likely present in Hawaii at least decades prior [13,14,15]. The Formosan subterranean termite has since spread to all other major islands [14], and is the most costly termite of Hawaii [16].

In 1963, the Asian subterranean termite, *C. gestroi* (previously referred to as *C. vastator* in early records in Hawaii) was first found in Hawaii at a single site in the neighborhood of Kaimuki in Honolulu, but has since become established in the vicinity of Ewa and Kalaeloa, Oahu, where it was collected during a 1988 to 1989 survey [17]. *C. gestroi* causes severe damage [17,18].

The West Indian drywood termite, *Cr. brevis*, is a global pest transported by commerce, and has been established in Hawaii before 1884 [13]. *Cr. brevis* is the second most costly termite species in Hawaii [16]. The only known natural (not occurring in human structures) infestation of *Cr. brevis* outside of its ancestral home of Peru and Chile [19] is in Nanakuli, Oahu [20].

The Pacific dampwood termite, *Zootermopsis angusticollis* (Hagen), has been found in high elevations on Maui [11]. *Z. angusticollis* is originally from the western coast of North America extending from Idaho, Montana and western Nevada [21]. *Z. angusticollis* is generally found in dead trees [21], and can infest structural wood [22].

*N. connexus* was described in 1923, but was recorded in Hawaii as early as 1883 [13]. It is considered to be a beneficial decomposer, and is found in higher elevations and in forests, as it requires higher moisture levels [23]. *N. connexus* has also been found around Polynesia, including the Society Islands and Marquesas Islands [24], and has likely been present in Hawaii for centuries [16].

*Incisitermes immigrans* (Snyder) is also said to have been present in Hawaii since before 1883 [13], also possibly for centuries [16]. It is distributed around Polynesia as well, including Washington Island, Fanning Island, Jarvis Island, Marquesas Islands, and also spans to the Galapagos Islands and Ecuador [24]. *I. immigrans* is also considered beneficial, and rarely infests buildings [13].

*Incisitermes minor* (Hagen), the Western drywood termite, is native to the southwest United States and northwest Mexico [25], but has spread sporadically through the United States and to Japan [26]. Infestations have also been reported in China and Canada [27,28]. *I. minor* causes severe structural damage and is responsible for most drywood termite damage in the western United States [29]. In Hawaii, it has been collected from only three locations on Oahu, but swarms have occurred over several years [30].

*Cryptotermes cynocephalus* Light was first recorded in Hawaii in 1996 [20]. It is known to occur in forests as well as structural lumber [20]. *Cr. cynocephalus* is native to Australia, and also occurs in Southeast Asian island nations, such as Indonesia and the Philippines [20].

Scheffrahn et al. [20] surveyed hand-collected termites on Oahu in 1996. Five species of termites: *C. formosanus*, *Cr. brevis*, *Cr. cynocephalus*, *I. immigrans*, and *N. connexus*, were found at eighteen coastal sites around Oahu [20]. Woodrow et al. [17] reported twenty-five sites of *C. formosanus* infestations from a 1998 to 1999 survey as well as six sites of *C. gestroi* infestations from 1999 to 2000. *C. formosanus* was found on the North Shore, the Windward coast, central Oahu, Honolulu, and surrounding Pearl Harbor [17]. *C. gestroi* was found in a limited range on the leeward coast, near Barber’s Point (now Kalaeloa) and in Ewa Beach [17].

### 1.2. Termite Swarming

Termite swarming occurs when adult reproductive termites (alates) exit the colony’s nest and search for a mate. Termite alates have compound eyes, sclerotized and pigmented exoskeletons, and four membranous wings of about equal size and shape [14,16]. Alates are often drawn to sources of light [14]. Evolutionarily “lower” termites tend to have small swarms under a wide range of environmental conditions, over a long season, while “higher” termites have larger swarms during specific environmental conditions, over a shorter season [31]. The most economically-important termite, *C. formosanus*, exhibits flight patterns of both lower and higher termites [31].

Termite alates were last formally surveyed on Oahu in 1969 to 1971 [31]. Higa and Tamashiro [31] found the general swarming period of *C. formosanus* to be April through July, with peak capture in May, with small numbers of alates found throughout the year. Microenvironmental factors (wind velocity, light intensity, soil temperature, and vapor pressure deficit) regulating *C. formosanus* flight were surveyed in 1980 by comparing flight and non-flight days during swarming [32]. Wind velocity was the key microenvironmental factor associated with *C. formosanus* flight, while the other factors (e.g., soil temperature) did not differ significantly during flight and non-flight days [32]; specifically, *C. formosanus* swarming only began when winds were below 3.7 km/h.

As part of the University of Hawaii Termite Project’s Educate to Eradicate program, partner teachers and students in public schools (citizen scientists) participated in alate light-trapping. The Educate to Eradicate program targets schools and adult continuing education programs to raise termite prevention and treatment knowledge in order to promote broad-scale termite control in Hawaii [33,34]. Citizen science projects allow for increased data collection, aid in answering scientific questions, provide educational outreach, and promote scientific literacy [35]. However, citizen science projects may require increased effort to overcome possible problems, such as varied skill levels of participants, and data accuracy [35]. These possible problems may be addressed by testing protocols and educational materials, training participants, and vetting data [35].

### 1.3. Objectives

To gather information on termite swarming on Oahu, light-traps were monitored at eight locations. In partnership with citizen scientists, termite swarming was monitored to (1) map the occurrence of termite alates across Oahu; (2) track variation in swarming over time; and (3) correlate swarming with environmental factors (temperature, precipitation, wind, and moon phase). Information on termite swarming phenology may contribute to management solutions focused on preventing or limiting new colony foundation [36] and to find the swarming patterns of recently established termites, such as economically important *C. gestroi*.

A hand-collection survey of termites on Oahu has not been carried out since 1998 to 2000 [17]. Hand-collection surveys may find newly-established species of termites; e.g., termite colonies must be mature to produce termite swarms. Hand-collection surveys can cover a larger area than light-trap surveys because they require less time. A current survey of termites may also aid in targeting locations for implementation of the University of Hawaii’s Termite Project’s Educate to Eradicate curriculum program to educate students and their parents/guardians about prevention of economically-important termites.

## 2. Materials and Methods

### 2.1. Swarming Survey

Light-traps were used to collect termite alates on Oahu, Hawaii, from February 2011 to September 2012.

#### 2.1.1. Locations

Eight locations (Table 3) on Oahu were monitored with light-traps either by the University of Hawaii Termite Project or by citizen scientists. Four of these locations were areas where seven field subterranean termite colonies, (University of Hawaii at Mānoa (*n* = 4), Kalaeloa (*n* = 1), Makakilo (*n* = 1), and Waimanalo (*n* = 1)), were located. Traps at these locations were monitored by the University of Hawaii Termite Project from February 2011 to September 2012. Students from Washington Middle School, Kaiser High School, and Mililani High School monitored traps at school (Washington Middle School, Honolulu, HI, USA) or at home (Kaiser and Mililani High School, Honolulu, HI, USA and Mililani, HI, USA, respectively) during the spring and summer months (March to May or August, 2011 to 2012). Citizens at a home in Aiea also monitored traps in 2011 and 2012.

Light-trap monitoring ended in September 2012 due to limited availability of staff and participants. Four additional locations were initially investigated but excluded, and individual light-traps were sometimes removed at different times during the study because of trap interference, construction, safety concerns, or availability of citizen scientists. Therefore, the number of light-traps per location varied.

Sites were mapped with Google Earth [37]. Students with take-home traps mapped their nearest cross-streets or their home if they had permission from parents. Students who did not provide reliable location information were excluded from distribution maps and statistical analyses.

#### 2.1.2. Light-Trap Construction

**Figure 1:**
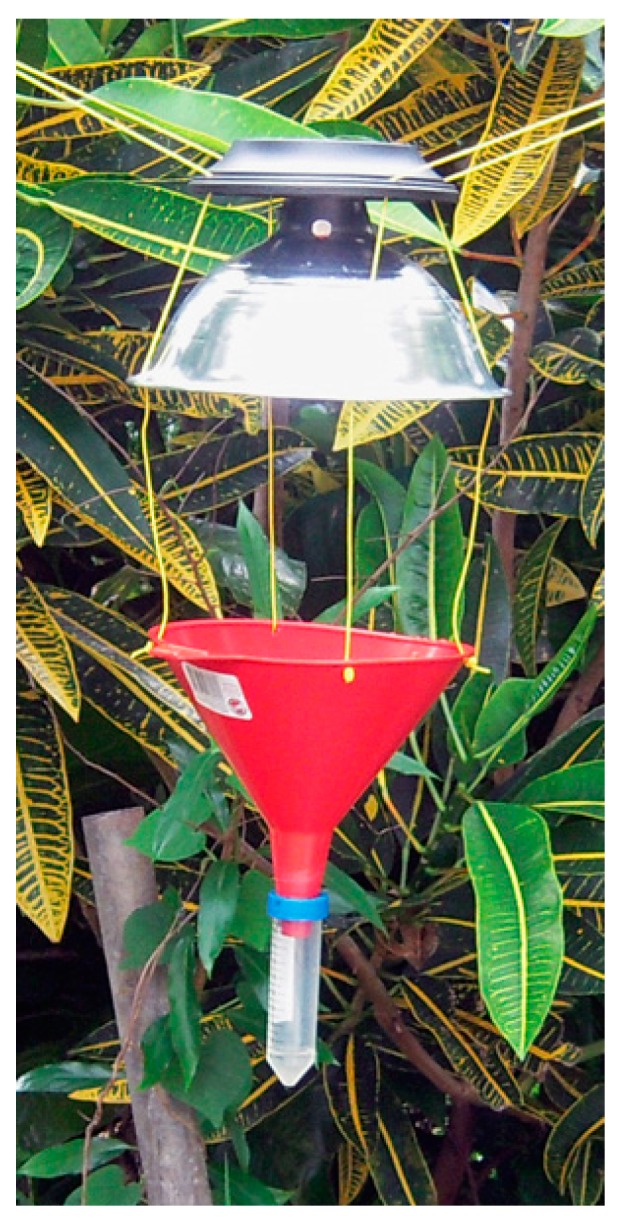
Light trap design. From top: solar light source (taken from landscape lights), aluminum reflector, plastic funnel, and removable centrifuge tube (made by coring and gluing the cap to the funnel tip).

Developed by Makena Mason, plastic funnels (model number F3005064, Blitz USA, Miami, OK, USA) were attached with string to aluminum reflectors (model number 758647, Southwire-woods, Chicago, IL, USA) and Hampton Bay solar LED landscape lights (model number NXT-74052, Home Depot, Atlanta, GA, USA) that automatically turned on after dark. Screw caps from 50 mL centrifuge tubes (model number 0644321, Fisher Scientific, Pittsburgh, PA, USA) were cored and glued to the funnels to allow the tubes to be twisted off regularly (Figure 1). Some light-traps were hung under existing lights (e.g., porch lights, hallway lighting); these traps did not use a hood or solar light source. Traps exposed to rain used centrifuge tubes with drainage holes (two pin-sized holes about 1.5 to 2 cm from the bottom of the tube). A few centimeters of water in the centrifuge tubes decreased the amount of beetle and desiccation damage to termite alate bodies. Citizen-scientists using solar light sources were instructed to hang light-traps at least 3 m away from competing sources of light, and to ensure solar lights were exposed to sunlight.

#### 2.1.3. Monitoring Light-Traps

Lab-monitored light-traps were checked bi-weekly for termite alates (weekly during winter months). Citizen scientist-monitored light-traps were checked every school day (Washington Middle School), every day (Kaiser High School, Mililani High School), or every other day (Miliani High School). Centrifuge tubes from traps with termite alates were removed and frozen. The date and trap code were recorded on the tube or on a paper inside the tube for later identification.

#### 2.1.4. Termite Alate Identification

Termite alate bodies and wings were counted and stored in 70% ethanol. The number of termites per trap was taken from the larger of either (number of termite bodies) or (number of termite wings divided by four, rounded up to a whole number). Termite alates were identified using a variety of resources [15,16,23,38,39]. Termites that could not be identified (e.g., mold, beetle, or physical damage, etc.) were omitted from analyses. Data from citizen scientists with unclear locations were also omitted from analyses.

#### 2.1.5. Swarming Analysis

Termite alate species occurrences were mapped using ArcGIS 10.1 [40]. The total number of termite alates per week for all locations was divided by the total number of light-traps out per week and averaged by month, then graphed with average temperature, rainfall, and wind speed [41].

Statistical analyses focused on *C. formosanus*, the most economically important termite. Locations had different numbers of light-traps; therefore, the total number of *C. formosanus* alates per location per week was divided by the number of light-traps per location to obtain the alate activity density.

To find peak swarming periods during the entire study, *C. formosanus* capture per location per week was averaged to find the average *C. formosanus* capture per location per month. Over half of the months had no *C. formosanus* captures and were excluded from the analysis. For the remaining months that had *C. formosanus* alates, average *C. formosanus* alate captures per location per month were transformed (√x + 0.5) and subjected to analysis of variance. Means were separated by Tukey’s Honest Significant Difference (HSD) using JMP [42] to find peak swarming periods.

To find relationships with environmental factors, *C. formosanus* data were first combined by week and location and square root-transformed. A multiple regression with average wind speed, rainfall, temperature, and percent moon illuminated as explanatory variables was then performed using JMP [42]. The multiple regression was limited to combined locations with *C. formosanus* alates, and only using data from first to last alate capture of the year per location. Weather data were taken from nearby weather stations [41]. Moon phase data were acquired from the Astronomical Applications Department of the U.S. Naval Observatory [43].

### 2.2. Hand-Collection Survey

A systematic hand-collection survey of termites was conducted on Oahu, Hawaii, from September to November 2012.

#### 2.2.1. Mapping of Points

A random starting point between 0 and 1000 m from the beginning of major roads (Farrington Highway, Kalanianaole Highway, Kamehameha Highway, Kaukonahua Road, King Street, Pali Highway, or Roosevelt Avenue) was selected. From this starting point, possible collection points were generated at 1 km intervals using “Construct Points” in ArcMap 10.1 [40]. Points were numbered (e.g., PPFARR01 for “Planned Point Farrington Highway 1”) and evaluated for suitability in Google Earth [37] street view. Suitable points were areas that were accessible (not on private property) and safe (not on a steep cliff or dangerously close to traffic).

Starting from the random point, every fourth point was selected (4 km intervals). Points considered unsuitable were replaced with the nearest suitable point (1 to 2 km away from original selection) or discarded entirely if no replacement was accessible. Points that fell within state parks where a permit was denied (Kaena Point) were also discarded.

#### 2.2.2. Collection

Special use application permits were obtained for collecting termites at points that fell within state parks from the Division of State Parks. No permits were necessary for city parks.

A timed search (one person, 30 min) for termites occurred at each suitable site. A 15 m tape measure was laid down starting from the edge of the pavement or edge of a barrier (e.g., stone wall or fence) in a perpendicular direction. Collection occurred within the 15 m distance. The area searched varied from 25 to 450 m^2^, depending on the layout of the site and searchable substrates; however, all searches were of the same duration (30 min).

Termites were collected from tree trunks and leaf litter. Fallen branches with evidence of termite damage (e.g., frass, kick-out holes, mud tubes, etc.) were opened to extract living termite soldiers, alates, and workers. Branches that were difficult to open were set aside, and extraction took place after the thirty minute search. Termites were collected by hand with an aspirator or forceps. Specimens were stored in 95% ethanol.

Latitude, longitude, and altitude were taken with a Garmin Global Positioning System (GPS) 76. Coordinates were then re-checked with Google Earth [37] for accuracy. Sites were labeled sequentially to get map codes.

Map layers were downloaded from the State of Hawaii’s Office of Planning Geographic Information System (GIS) Data site, the U. H. Geography Department, and the United States Department of Agriculture Natural Resources Conservation Service’s Geospatial Data Gateway [44,45,46]. Average rainfall (mm) and elevation contours (10 m) were used to create maps and spatially join data to points. Layers available as raster data were first converted to points and spatially joined with ArcMap 10.1 [40]. Vegetation zones were mapped with ArcMap 10.1 [40] using Ripperton and Hosaka’s map [9] as an overlay.

#### 2.2.3. Analysis

Termites were identified based on the soldier or reproductive caste using a variety of resources [15,16,23,38]. A mandibular comparison with known species was used for a site where only drywood termite nymphs were found. Termite occurrences were mapped using ArcMap 10.1 [40]. Abiotic factors (elevation, average annual rainfall, vegetation zone, and physiographic (windward/leeward) zone) from a variety of sources [9,44,45,47] were spatially joined to site point data using ArcMap 10.1 [40]. Termite alate occurrences were compared with hand-collection survey occurrences. A generalized linear model (binomial, logit link) using JMP [42] was performed on the presence or absence of *I. immigrans* with elevation and average annual rainfall, since it was the only species found at more than two sites.

## 3. Results and Discussion

### 3.1. Swarming Survey

**Figure 2:**
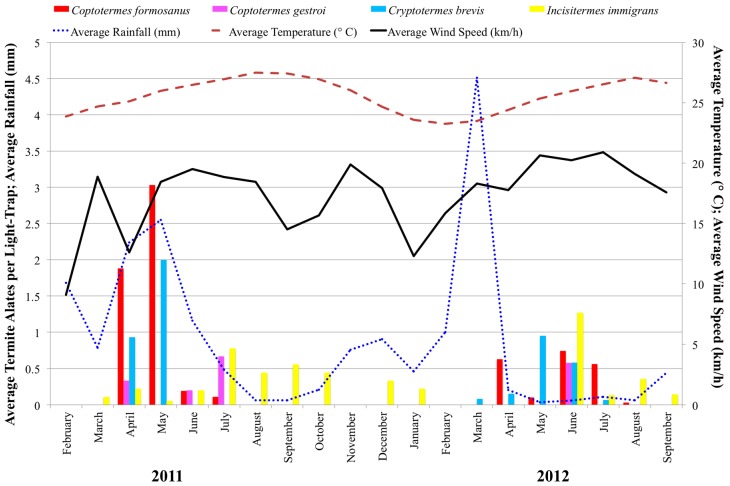
Average termite alate activity density from light-traps on Oahu, Hawaii, from February 2011 to September 2012. Average rainfall (mm), temperature (°C), and wind speed (km/h) from the Honolulu International Airport weather station [41].

Termite alates were found every in month except February 2011, November 2011, and February 2012 (Figure 2). *C. formosanus* alates were captured between April and July 2011 and April and August 2012 from Aiea, Mililani, UH Mānoa, Waimanalo, and Washington Middle School (Figure 2).

In months during which *C. formosanus* alates were captured, a two-factor analysis of variance using combined location and month revealed capture rates differed by month (*F*_8,11_ = 6.43, *p* < 0.003), but not by location (*F*_4.15_ = 1.16, *p* < 0.367). Post-hoc Tukey’s HSD showed the peak month of capture to be May 2011 (Table 4). Higa and Tamashiro [31] found similar peaks in swarming during 1969 and 1970 with large numbers of *C. formosanus* alates swarming from April to July.

The results of the multiple regression indicated that environmental factors predicted *C. formosanus* alate capture (*R*^2^ = 0.29, *F*_4,47_ = 4.58, *p* < 0.0025). Average wind speed (*β* = −3.68, *p* < 0.0006) and average rainfall (*β* = 2.20, *p* < 0.0325) significantly predicted *C. formosanus* alate activity density, while average temperature and percent moon illumination did not (Table 5a). Formosan subterranean termite swarming correlations with environmental factors are consistent with previous findings on Oahu that found wind velocity to be a key environmental factor regulating swarming [32] (Table 5b). Leong et al. [32] found termite swarming to start when winds were below 3.7 km/h. However, Leong et al. [32] used direct measurements, while the design of the present study used average wind speed per week, as termite alates were collected and combined by week. Despite these differences in methodology, wind velocity remains a predictive environmental factor for *C. formosanus* swarming. When differences in wind speed are accounted for, average rainfall becomes a significant predictor of *C. formosanus* alate capture. *C. formosanus* termites’ high susceptibility to desiccation [14] may explain the positive relationship of *C. formosanus* capture with rainfall. Bess [13] noted *C. formosanus* swarmed on “humid days, often immediately followed by a shower”. Further, if rainfall is too high, it may also be detrimental to *C. formosanus* [31], which may contribute to the lower number of termite alates in 2012 compared to 2011.

Other factors than those tested in a multiple regression may influence the observed variable, possibly in a manner that correlates the observed variable to its past and future values, known as autocorrelation. Autocorrelation may inflate *t*-values by decreasing the standard errors between time periods and overestimate the explanatory power of the multiple regressions’ factor(s). The Durbin-Watson statistic assessing serial autocorrelation was 1.67 (*p =* 0.0697). A post-hoc comparison of the same regression of environmental factors to *C. formosanus* alate activity density by trap monitor (citizen scientists vs. the termite lab) revealed differences in the Durbin–Watson statistic: D–W = 2.96 (*p =* 0.9371) for citizen scientist-monitored traps, indicating no significant serial autocorrelation, and D–W = 1.01 (*p <* 0.0004) for termite lab-monitored traps, indicating significant serial autocorrelation, which violates an assumption of the multiple regression analysis, which could be due to the study design. Light-traps for lab-monitored locations were hung above known *C. formosanus* colonies, increasing the likelihood of consistent, and possibly auto-correlated, alate activity density. Another possible explanation for autocorrelation is the emergence pattern of *C. formosanus* alates, in which alates emerge slowly over a period of three weeks or longer [32]. Citizen scientist-monitored traps had a statistically insignificant Durbin-Watson statistic for autocorrelation; combination with lab data, therefore, decreased the multiple regression model’s likelihood for autocorrelation.

*C. formosanus* alate activity density in the summers of 2011 and 2012 were not similar. Average wind speed during swarming was 9.44 km/h (s.d. = 3.95) in 2011 and 16.81 km/h (s.d. = 4.89) in 2012, which may contribute to lower numbers of *C. formosanus* alates observed in 2012. Other environmental variables, such as temperature, or intrinsic factors that influence *C. formosanus* flight may also account for swarming variability. For example, in New Orleans, termite swarming showed a pause between swarming months, possibly due to developmental times [48].

The height of light-traps may also influence the number of termite alates caught in light-traps. Lab-monitored traps were from 1.5 to 3 m high, with most about 2 m high. However, this variable was not recorded; further, it is not known at what height students hung traps, though it may be assumed they were within 1 to 3 m. The “PUB” light-trap from UH Mānoa was hung from a tree with a string-pulley that raised the trap to about 3 m. This trap also had the highest number of alates in a single week (*n* = 33). However, there were no significant differences between *C. formosanus* activity densities at different locations.

Data from the current study may provide a baseline for studies measuring termite suppression and changes in termite distributions on Oahu. However, studies comparing future swarming data over time should account for wind speed and rainfall, as these were significant predictors of *C. formosanus* alate capture.

**Figure 3:**
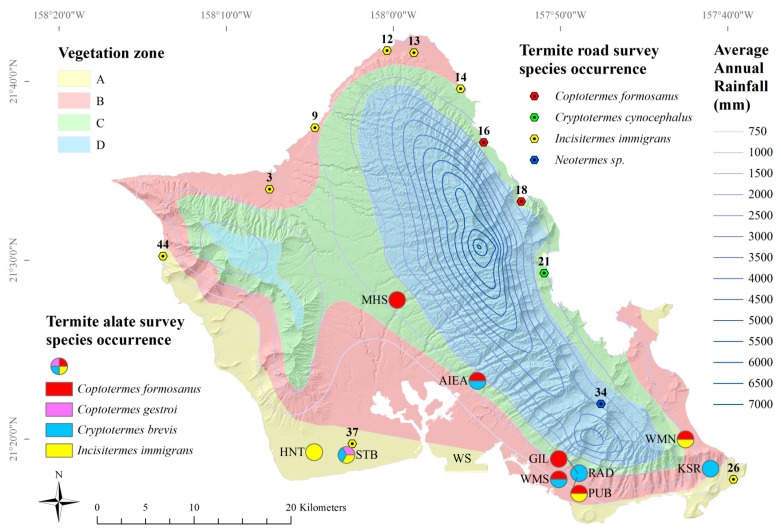
Termite occurrences for survey sites (by map code) and alate survey sites (by trap code) by vegetation zone [9] and average annual rainfall [45]. (NAD 1983 4N, WGS 1984). WS: Honolulu International Airport weather station; Light-trap codes: AIEA: Aiea; GIL: Gilmore Hall (University of Hawaii at Mānoa); HNT: Hornet (Makakilo); KSR: Kaiser High School; MHS: Mililani High School; PUB: Publication (University of Hawaii at Mānoa); RAD: Radiator (University of Hawaii at Mānoa); STB: Stables (Kalaeloa); WMN: Waimanalo Research Station (Waimanalo); WMS: Washington Middle School.

Asian subterranean termite, *C. gestroi*, alates were observed in April, June, and July 2011 (*n* = 14) and in June of 2012 (*n* = 8). *C. gestroi* was found only in Kalaeloa (Figure 3). West-Indian drywood termite, *Cr. brevis*, alates were captured in April to June 2011 and April to July 2012. *Cr. brevis* alates were found in Aiea, Kalaeloa, UH Mānoa, and Kaiser High School (Figure 3). *Cr. brevis* alates were captured primarily by citizen scientists, so the full range of swarming may not have been observed in 2011. Lowland tree termite, *I. immigrans*, alates were observed every month except February and November in 2011 and from June through September in 2012. *I. immigrans* alates were found in Kalaeloa, Makakilo, UH Mānoa, and Waimanalo (Figure 3).

Indo-Malaysian drywood termite, *Cr. cynocephalus*, western drywood termite, *I. minor*, and Pacific dampwood termite, *Z. angusticollis*, alates were not observed in light-traps. A single desiccated *Neotermes* sp. was found in a Mililani High School student’s light-trap, and is assumed to be the forest tree termite, *N. connexus*, as it is the only species of this genus known from Hawaii, although its species could not be identified.

In addition to decreasing the autocorrelation in the multiple regression, citizen scientists expanded the geographic range of the study and increased the sample size. Participating teachers found the project a useful addition to the curriculum. Some students were able to use their data for further questions for science or senior projects. Light-trapping termites contributed to scientific literacy, as students gathered data, read scientific literature, and were taught to differentiate drywood and subterranean termite alates.

Not all species of termites that occur in Hawaii were found in light-traps. Future studies may include: continued alate trapping in current areas, alate trapping in new areas, and trapping near known locations of *Cr. cynocephalus*, *N. connexus*, and *Z. angusticollis* to better define these species’ swarming phenologies on Oahu. The Western drywood termite, *I. minor*, may not be attracted to light-traps, as they swarm during the day. The geographic range of the study was also limited, with no sites on the north or west portions of Oahu, and these areas may be targeted for future alate surveys. For *Cr. brevis*, light-trapping may even be a control strategy, as it can decrease the number of dispersing alates [49].

### 3.2. Hand-Collection Survey

**Figure 4:**
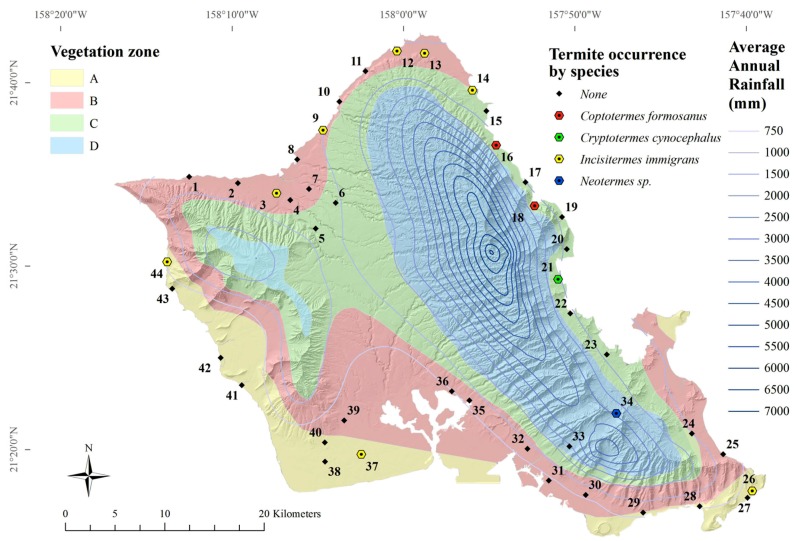
Termite occurrence by vegetation zone [9] and average annual rainfall [45] (NAD 1983 4N, WGS 1984).

Four species of termites were found from 44 roadside sites on Oahu, Hawaii (Figure 4). The lowland tree termite, *I. immigrans*, was most frequently encountered (*n* = 8), followed by the Formosan subterranean termite, *C. formosanus* (*n* = 2) (Table 6). The Indo-Malaysian drywood termite, *Cr. cynocephalus*, was found at one site (Table 6). A termite likely from the genus *Neotermes* was found at one site (Table 6), and only *Neotermes connexus* is known from this genus in Hawaii. *C. gestroi*, *Cr. brevis*, *I. minor*, and *Z. angusticollis* were not found at any road survey site. Abiotic factors by species occurrences are given in Table 7.

The lowland tree termite, *I. immigrans*, was the most frequently encountered termite, consistent with the findings of Scheffrahn et al. [20]. *I. immigrans* termites were found at around the same areas along the North Shore, Waianae Coast and down to Malaekahana State Park along the Windward Coast, although they were absent from several areas previously identified along the Windward coast [20]. *I. immigrans* termites were found near alate light-traps that captured *I. immigrans*, *C. gestroi*, and *Cr. brevis* (Figure 3). *I. immigrans* alates were found in four out of ten light-trap locations (Figure 3). *I. immigrans* was not significantly affected by average elevation or average annual rainfall (GLM, *F* = 4.61, *p* < 0.10).

The two road survey occurrences of *C. formosanus* were not found near alate light-trap locations (Figure 3). *C. formosanus* alates were found in six out of ten light-trap locations (Figure 3). Formosan subterranean termite, *C. formosanus*, workers and soldiers were found crawling freely (not in mud tubes) along the base of a tree in Hauula and in a tree stump in Ahupuaa O Kahana State Park. Scheffrahn et al. [20] found *C. formosanus* colonies near the same area, but further south, along Kaneohe Bay, as well as Waahila Ridge, Heeia, Kahaluu, and Punaluu. The previous study [20] also found *N. connexus* termites where the current study found *C. formosanus*. This may be because of both species’ relatively high moisture requirement. *C. formosanus* distributions may further be delimited with increased light-trapping on the north and west sides of Oahu. It is not clear whether *C. formosanus* is actually decreasing in range or if the current survey methodology resulted in fewer findings. However, years of termite education, prevention measures (e.g., Basaltic Termite Barrier, Termi-mesh, termite-resistant wood, etc.), bait-systems and other chemical controls may have contributed to smaller populations of *C. formosanus*.

*Cr. brevis* alates were found at half of all light-trap sites (Figure 3); however, *Cr. brevis* termites were not found at any road survey location. This is not unexpected, since this species very rarely occurs in natural settings, although known to be well-established in structures throughout the island. *Cr. brevis* was extremely common in alate light-traps (Figure 3). Alate trapping is, therefore, recommended to study distributions of *Cr. brevis*.

The Indo-Malaysian drywood termite, *Cr. cynocephalus*, was found in fallen logs near a stream between the two sites at which it was first found in 1996: Waiahole Valley Road and Kualoa Regional Park [20]. Several nymphs were found, along with alates, indicating a mature colony. *Cr. cynocephalus* termites were not found at any surrounding survey sites, which may mean its dispersal is still somewhat limited.

The Western drywood termite, *I. minor*, was not found at any sites, possibly because it has yet to expand its range, or because it remains in structures that were not examined. *I. minor* swarms during the day, and may not be attracted to light-traps. Incidental reports may be the best method at present for locating *I. minor* infestations. Pacific dampwood termite, *Z. angusticollis*, was not found in this survey. Currently, its known distribution remains limited to Kula, Maui. Survey sites in this study were mostly at low elevations near coastlines. Tree branches with evidence of termites were found at multiple locations, but live termites were not always found, especially with regard to subterranean termites. Subterranean termites are cryptic, and light-trap surveys may be more effective in mapping their distribution than hand-collection techniques. Crowd sourcing, or gathering data from the public on occurrences of termites may also be an option, and pest control reports may also be useful.

At some locations, soldiers and imagoes (alate and dealate reproductives) were not found. At the Pali lookout site (map code 34), two pairs of dealate reproductive termites were found, but positive identification was difficult because of the lack of wings and absence of any soldiers. These dealates were darker, had a smaller pronotum width, a proportionately wider head to pronotum ratio, and larger eyes than voucher *N. connexus* alates. Further surveys in the area may reveal soldiers and more imago samples for positive identification.

## 4. Conclusions

Although no novel termite species were identified in these survey efforts, these data provide insight on the swarming period of *C. gestroi* in Hawaii and the environmental factors related to termite swarming, the localized spread of *Cr. cynocephalus*, and the continuing occurrence of established termite species on Oahu.

Updated data on economically important *C. formosanus* swarming show higher wind speeds negatively affect its capture, in agreement with previous studies [32]. Whereas previous studies [32] found rainfall to be an insignificant predictor of *C. formosanus* capture, the present study found it to be positively correlated. Studies investigating *C. formosanus* swarming over time should take wind speed and rainfall into account. In Hawaii, *C. gestroi* appears to swarm about a month later than peak *C. formosanus* swarming.

## Figures and Tables

**Table 1:** Vegetation zones of Hawaii as characterized by Ripperton and Hosaka [9].

Zone	Elevation (m)	Average Annual Rainfall (mm)	Vegetation
A	0 to 600 (usually <150)	<500	Xerophytic shrubs (mostly exotic), coastal fringe of trees
B	0 to 900 (average of 600)	500 to 1000	Xerophytic shrubs, trees in higher elevations
C	0 to 1200	1000 to 1500	Open shrubs and grasses in lower elevations, mixed open forest
D	0 to 2000	>1500	Shrub to open grasslands and closed and open forests
E	>2000	<1270	Open forest and shrub, upland forest to above tree line (Hawaii and Maui islands only)

**Table 2:** Termite species of Hawaii.

Family	Species	Common Name
Termopsidae		
	*Zootermopsis angusticollis* *	Pacific dampwood termite
Kalotermitidae		
	*Neotermes connexus*	Forest tree termite
	*Incisitermes immigrans*	Lowland tree termite
	*Incisitermes minor* *	Western drywood termite
	*Cryptotermes brevis* *	West-Indian drywood termite
	*Cryptotermes cynocephalus* *	Indo-Malaysian drywood termite
Rhinotermitidae		
	*Coptotermes formosanus* *	Formosan subterranean termite
	*Coptotermes gestroi* *	Asian subterranean termite

* Structure-destroying pests. Others may occasionally be found in structural lumber.

**Table 3:** Number of light-traps per location on Oahu, Hawaii. For light-traps monitored by citizen scientists (CS), only those with specific locations are included in the count.

Location/Coordinates (Decimal Degrees)	Monitor	Date Range (Month/Year)	Traps Per Location	
			N (2011)	N (2012)
Aiea/21.3880, −157.9165	CS	4/2011; 3/2012–9/2012	1	1 to 2
Kaiser High School/21.2851, −157.6945	CS	3/2011–5/2011	3	
Kalaeloa/21.3192, −158.0465	Lab	2/2011–9/2012	3	3 to 6
Makakilo/21.3216, −158.0789	Lab	2/2011–4/2011	1	
Mililani High School/21.4535, −158.0091	CS	3/2012–9/2012	-	8
University of Hawaii at Mānoa/21.2979, −157.8174	Lab	2/2011–9/2012	2 to 4	3
Waimanalo/21.3334, −157.7098	Lab	2/2011–9/2012	4	
Washington Middle School/21.2961, −157.8357	CS	3/2011–5/2011; 3/2012–5/2012	3	2

**Table 4:** Least squares means of *C. formosanus* alate capture from a two-factor analysis of variance for five locations by month. Levels not connected by the same letter are significantly different.

Month	Mean
May 2011	4.27 ^a^
July 2012	3.08 ^a,b^
April 2011	2.96 ^a,b^
April 2012	2.07 ^a,b^
June 2012	1.58 ^b^
July 2011	1.23 ^b^
June 2011	1.20 ^b^
May 2012	1.16 ^b^
August 2012	1.00 ^b^

**Table 5:** (**a**) Multiple regression results for environmental factors with *Coptotermes formosanus* alate activity density from light-traps on Oahu, Hawaii; *: significant at *p* < 0.05, **: significant at *p <* 0.001; (**b**) Previous and current study environmental associations with *C. formosanus* alate capture on Oahu. NA: not applicable, NS: not significant.

**(a)**			
**Environmental Factor**	***β***	***F* Ratio**	***p*-Value**
Mean temperature (°C)	1.75	3.06	0.2602
Mean wind speed (km/h)	−3.68	13.53	0.0006 **
Mean rainfall (mm)	2.20	4.86	0.0325 *
Mean percent moon illuminated	0.22	0.05	0.8234
**(b)**			
**Environmental Factor**	**Higa and Tamashiro 1983**	**Leong et al. 1983**	**Current Study**
Mean temperature (°C)/Soil temperature	NS	NS	NS
Mean wind speed (km/h)	NA	Significant	Negative correlation
Mean rainfall (mm)	NS	NA	Positive correlation
Mean percent moon illuminated/Light intensity	NA	NS	NS
Humidity	NS	NA	NA
Vapor pressure deficit	NA	NS	NA

**Table 6:** Termite species occurrence (N) on Oahu, Hawaii by island zone [47] and vegetation zone [9]. L: leeward; W: windward.

Family	N	Frequency (*n* = 44)	Island Zone (N)		Vegetation Zone (N)			
Species			L	W	A	B	C	D
Kalotermitidae								
*Incisitermes immigrans*	8	0.18	5	3	3	4	1	0
*Cryptotermes cynocephalus*	1	0.02	0	1	0	0	1	0
*Neotermes* sp.	1	0.02	0	1	0	0	0	1
Rhinotermitidae								
*Coptotermes formosanus*	2	0.05	0	2	0	0	2	0
**Total**	12		5	7	3	4	4	1

**Table 7:** Elevation (m) and average annual rainfall (mm) means and standard deviations by species occurrences at N sites (total sites = 44).

Family		Elevation (m)		Average Annual Rainfall (mm)	
Species	N	Mean ± Std. Dev.	Range	Mean ± Std. Dev.	Range
Kalotermitidae					
*Incisitermes immigrans*	8	5 ± 4.24	1 to 12	896 ± 231.19	544 to 1246
*Cryptotermes cynocephalus*	1	1	1	1586	1586
*Neotermes* sp.	1	362	362	2454	2454
Rhinotermitidae					
*Coptotermes formosanus*	2	1 ± 0.004	1 to 1	1700 ± 94.97	1633 to 1767
**Total**	12		1 to 362		544 to 2454
